# Antenatal education for labour and postpartum pain: A scoping review of content, delivery approaches, evidence gaps, and lived experiences

**DOI:** 10.1371/journal.pone.0330399

**Published:** 2026-06-03

**Authors:** Elliot Sloyan, Elizabeth Leddy, Carol Clark, Sinéad Dufour, Rosie Harper, Alex Dunford, Ömer Elma

**Affiliations:** 1 School of Allied Health and Exercise Sciences, Faculty of Health, Environment and Medical Sciences, Bournemouth University, Bournemouth, United Kingdom; 2 Pain Science Research Group, Centre for Wellbeing and Long-Term Health, Bournemouth University, Bournemouth, United Kingdom; 3 School of Rehabilitation Science, Faculty of Health Sciences, McMaster University, Ontario, Canada; 4 University Hospitals Dorset NHS Foundation Trust, Poole, Dorset, United Kingdom; 5 Pain in Motion Research Group (PAIN), Department of Physiotherapy, Human Physiology and Anatomy, Faculty of Physical Education and Physiotherapy, Vrije Universiteit Brussel, Brussels, Belgium; Texas Woman's University - College of Nursing, UNITED STATES OF AMERICA

## Abstract

**Background:**

Pain during labour and the postpartum period is a complex and multidimensional experience. Antenatal education programmes aim to prepare individuals for childbirth and early parenthood; however, the extent to which these programmes address labour and postpartum pain management, and how women experience this education, remains unclear. This scoping review aimed to map the content, delivery characteristics, and evidence gaps of antenatal education programmes addressing labour and postpartum pain, including women’s lived experiences.

**Methods:**

This review was conducted in accordance with PRISMA-ScR and Joanna Briggs Institute guidelines. The protocol was registered with the Open Science Framework (6597j). Twelve electronic databases were systematically searched in November 2025. Quantitative, qualitative, and mixed-methods primary studies examining antenatal education programmes with a focus on labour or postpartum pain were included. A narrative synthesis was undertaken to map intervention content, delivery approaches, and pain-related outcomes and experiences.

**Results:**

A total of 5,959 records were identified from the search strategy. A total of 17 articles met the eligibility and inclusion criteria, including seven randomised controlled trials, seven quasi-experimental studies, one pre-post study, and two qualitative studies. The content and structure of antenatal education interventions between studies was heterogenous. Common themes included the distinction between “true and false labour pain” and breathing exercises. Qualitative findings highlighted women’s perceived improvements in pain coping, confidence, sense of control, and use of non-pharmacological strategies during labour.

**Conclusion:**

Antenatal education programmes contain limited information on labour and postpartum pain management, with little consistency across interventions. While non-pharmacological strategies appear valuable in supporting coping and confidence during labour, pain mechanisms and postpartum pain remain under-addressed. Incorporating pain-focused education such as pain neuroscience principles may enhance antenatal education and support more effective pain management. Further research is required to develop and evaluate consistent, evidence-based antenatal education approaches that address both labour and postpartum pain.

## Introduction

Women’s experience of pain during labour and the postpartum period is a complex and multifactorial experience influenced by physiological, psychosocial, and cultural factors [1]. Although labour pain is often functional and indicative of progress, it is typically intense and can provoke fear, loss of control, and emotional distress [2]. When poorly managed, labour pain is associated with a range of adverse maternal outcomes, including prolonged labour, increased obstetric interventions, postnatal depression, persistent postpartum pain, and birth trauma [3–6]. As many as 90% of birthing women globally report experiencing pain they describe as “severe” or “very severe” [7]. Pain intensity and suffering are distinct, with suffering reflecting the emotional dimension of pain, while intensity often refers to the magnitude of the sensation [8]. Psychological factors, such as fear of childbirth and pain catastrophising, are linked to heightened pain perception, greater reliance on pharmacological interventions, and a higher incidence of traumatic birth experiences with long term impact [6,9,10].

Antenatal education has become a key feature of pregnancy care, providing expectant parents with knowledge and skills to prepare for labour, birth, and early parenthood [11]. These programmes vary in delivery format and content but often include topics such as stages of labour, birth planning, breastfeeding, new-born care, and partner involvement. Whilst pain management strategies are included [12] there are inconsistencies in how pain education is delivered [13,14]. Recognising the value of preparation for birth, the World Health Organisation (WHO) recommends antenatal education as an essential component of pregnancy care [15]. However, guidance from regulatory bodies such as the National Institute for Health and Care Excellence (NICE) has yet to mandate consistent inclusion of pain education within antenatal care [16]. There remains an absence of standardisation in antenatal education content, with many programmes lacking comprehensive guidance on managing labour and postpartum pain through conservative means such as pain education and mindfulness strategies, despite a growing body of evidence showing the psychological benefits of antenatal education interventions [17,18].

In the United Kingdom, the absence of national standards or defined curricula results in wide variation in whether, and how, pain is addressed in antenatal education contributing to the “postcode lottery” of care, where the availability and quality of education depend on geographical location, thereby widening health inequalities [6,14]. In addition, the variability in educational provision also contributes to unequal birth experiences and potential over-reliance on pharmaceutical interventions, including epidurals and opioids, which carry associated maternal and foetal health risks [5,19]. This gap limits opportunities to shape maternal expectations, reduce fear, promote self-efficacy, and support informed decision-making around labour pain management.

Overall, understanding the characteristics and scope of existing antenatal education may help clarify how education addresses pain during labour and in postpartum recovery. Strengthening conservative approaches to pain management across pregnancy, labour and birth has the potential to improve maternal and foetal outcomes and reduce reliance on pharmacological interventions. Accordingly, the aim of this scoping review is to map and synthesise the content, delivery approaches, and scope of antenatal education programmes that address labour and postpartum pain; including women’s lived experiences of participating in these programmes. This review is guided by the following research questions: (1) What types of antenatal education programmes include content related to labour and postpartum pain management? (2) What pain-related content, educational approaches, delivery formats, and dosages are reported within these programmes? (3) What pain-related outcomes, experiences, and mechanisms do these programmes seek to influence or describe? and (4) What are women’s reported experiences and perceptions of antenatal education in relation to labour and postpartum pain? The objectives of this review are to identify and categorise antenatal education programmes that address pain, examine their core educational components and modes of delivery, synthesise both outcome-focused and experiential evidence, and identify gaps in the existing literature to inform future research and the development of more consistent, evidence-informed antenatal education practices.

## Methods

This scoping review was conducted in accordance with the Preferred Reporting Items for Systematic Reviews and Meta-Analyses Extension for Scoping Reviews (PRISMA-ScR) (**Please see** S1 Checklist) [20,21]. Resources to help define research questions and aims from the Joanna Briggs Institute (JBI) were utilised [22]. The protocol for this scoping review was registered with the Open Science Framework (6597j) prior to the review being conducted (15^th^ of November 2025).

### Search strategy

Prior to conducting the database search, an initial search of JBI Evidence Synthesis database and the Cochrane Database of Systematic Reviews was undertaken to identify any existing or ongoing scoping or systematic reviews addressing antenatal education for labour and postpartum pain. No current or recently published reviews with the same focus were identified, confirming the originality and relevance of the present review.

The following electronic bibliographic databases were systematically searched in November 2025 via EBSCOhost: CINAHL, Academic Search, PsycInfo, Education Source, SPORTDiscus, PsycArticles, and SocINDEX. In addition, PubMed, MEDLINE, Web of Science, and Cochrane CENTRAL. The search strategy was structured around the PICO/PECO framework, and the key search terms are presented in **Table 1**. The full search strategy, including Boolean operators, filters, and limits applied, is presented in **Table 2**.

**Table 1:** Search terms and PICO/PECO framework.

PICO Concept	Search terms
Population	Pregnant women
Intervention/Exposure	parent* education OR parent* preparation OR childbirth education OR childbirth preparation OR birth education OR birth preparation OR antenatal education OR prenatal education or antenatal preparation OR prenatal preparation
Comparison	With or without
Outcome measure	Pain

**Table 2:** Search strategy.

S1 (title search)	T (parent* education OR parent* preparation OR childbirth education OR childbirth preparation OR birth education OR birth preparation OR antenatal education OR prenatal education OR antenatal preparation OR prenatal preparation)
S2 (abstract search)	AB (parent* education OR parent* preparation OR childbirth education OR childbirth preparation OR birth education OR birth preparation OR antenatal education OR prenatal education OR antenatal preparation OR prenatal preparation)
S3 (title search)	T (pain)
S4 (abstract search)	AB (pain)
S5	S1 OR S2
S6	S3 OR S4
S7	S5 AND S6

**Abbreviations:** S, Search; T, Title only search; AB, Abstract only search

### Study selection and eligibility criteria

The study selection process was completed by two independent and blinded authors (ES and EL) using Rayyan software. Duplicate studies were identified and removed before screening. First, the two authors independently screened all titles and abstracts against eligibility criteria. Then, the full texts of articles that were retrievable were screened independently by the same authors. Discrepancies were resolved through discussion with the third and fourth researchers (OE and AD). The search strategy also included forward and backward tracking. Forward tracking involved looking for eligible studies that cited the included studies. Backward tracking involved searching the reference lists of the eligible studies.

No study was excluded based on publication date. This scoping review considered primary research studies of any methodological design, including quantitative, qualitative, mixed-methods, and observational studies, that examined antenatal education programmes addressing pain during labour, or the postpartum period among nulliparous and/or multiparous women

The exclusion criteria were as follows: protocol-only publications, conference abstracts without full text, feasibility or pilot studies without sufficient intervention description, studies with insufficient detail on antenatal education content, male-only populations, and studies where pain was not a reported outcome or focus.

### Data extraction and synthesis

Overall, data extraction focused on the contents and characteristics of the antenatal education interventions listed in the captured studies, as well as pain-related outcomes measures. Our data extraction tables were developed based on an iteration of a JBI extraction tool, which the screening process helped inform. For the interventional and observational studies, the extracted data consist of author, year of publication, country, clinical setting, study methodology, key findings, antenatal education content, frequency of education sessions, education delivery method, which profession delivered sessions, pain outcomes measured. Missing or supplementary data was acquired by contacting the study authors as required. For qualitative and mixed-methods studies, data relating to women’s experiences, perceptions, and reported use of antenatal education in relation to labour and postpartum pain were extracted where available and summarised as pain-related themes and key findings.

## Results

### Study selection and characteristics of included studies

**Fig 1:**
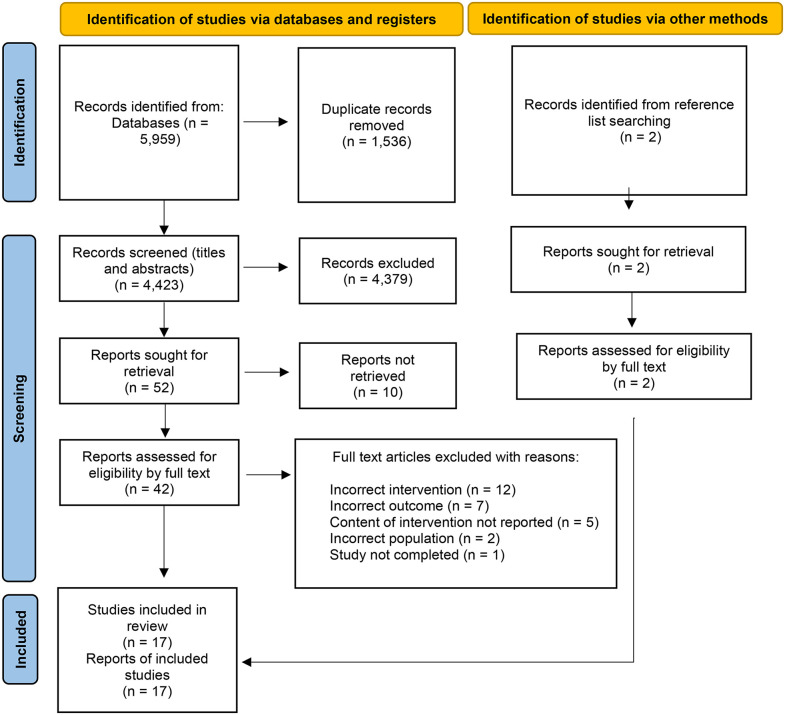
PRISMA flow diagram.

The details of the process of selecting studies in accordance with PRISMA-ScR standards can be found in **Fig 1**. A total of 5,959 studies were retrieved and screened against the inclusion and exclusion criteria. A total of 17 studies met the inclusion criteria, including seven randomised controlled trials (RCTs) [23–29], seven quasi-experimental studies [30–36], one pre-post-trial [37], and two qualitative studies [38,39].

The included studies were published between 2008 and 2025 and spanned populations from seven countries: Iran, Turkey, Thailand, Egypt, Hong Kong, Brazil, and the United States. Sample sizes ranged from 30 [26] to 832 [29]. A comprehensive overview of the characteristics of the selected studies can be found in Table 3 and Table 4.

**Table 3:** Data extraction table for interventional and observational studies.

Author; Year; Country; Study Design	Participants	Antenatal Education Content	Delivery Method; Frequency; Profession	Control Group	Outcome measure	Findings
Tomyabatra, 2019, Thailand, RCT	Intervention N = 432 Control N = 400	**Antenatal education via social networking on mobile phone** Video of serious complications including pain, vaginal bleeding, water breaking, fewer foetal movements. Distinction between true and false labour pain, maternity role, physical, mental changes, ominous signs to see doctors, sanitation, medication, counting of foetal movement, delivery preparation, alteration of the body near delivery, steps of delivery service in the hospital, a method of delivery, and breathing exercise for labour pain relief.	Audio-video media, social networking application, mobile phone Online, mobile phone plus routine ANC “Four times monthly and four times every two weeks” Registered Nurse running ANC	Routine antenatal group-health education Two-time standard ANC group-health education provided by a registered nurse with with information about maternity role, physical, mental changes, ominous signs to see doctors, sanitation, and medication. At 32 weeks’ gestation about the counting of foetal movement, delivery preparation, alteration of the body near delivery, the distinction between true and false labour pain, abnormal symptoms to see doctors, steps of delivery service in the hospital, a method of delivery, and breathing exercise for labour pain relief.	**Labour pain duration** Minutes	Significant difference in labour pain duration before admission between the intervention and the control groups (121.7 ± 95.3 versus 139.2 ± 7.0 minutes), coefficient –17.7 (95% CI –31.4 to –4.0); p = 0.011.
Duncan et al. 2017, USA, RCT	Intervention N = 15 Control N = 15	**The mind in labour (MIL): working with pain in childbirth** Mindfulness strategies for coping with labour-related pain and fear are taught through interactive, experiential activities, with periods of didactic instruction.	F2F 18 hours over three days ‘Mindfulness-Based Childbirth and Parenting Program (MBCP)” instructors	Standard childbirth education courses comparable in length and quality to the MIL intervention but without mindfulness, yoga, or other mind/body components (e.g., hypnosis). A list of approved courses was compiled via a web search and provider follow-ups. If no suitable option was available, participants could submit a preferred course for screening. TAU participants were encouraged to attend with a partner or support person; in this study, 12 reported doing so.	**Retrospective perceived labour pain intensity** VAS 10 cm line “No pain” to “worst possible pain” **Pain catastrophising scale** Rate degree to which they experience particular thoughts and feelings (e.g., “It’s awful and I feel that it overwhelms me” on a scale of 0 (Not at all) to 4 (All the time)	Condition was marginally associated with pain, with higher scores for MIL participants (average score of 5.20 on the 1–10 VAS) than for control participants (average score of 3.88; b = 1.32, p = .07). When condition was entered in a model with the four covariates, its estimated effect dropped slightly (b = 1.08, p = .14) and the model overall was nonsignificant (Multiple R2 = .29, p = .15) PCS dropped by 3.6 points in the MIL group and was essentially unchanged in the TAU group. The time*-group interaction was not significant (t = −1.06, p = .30; estimated treatment effect = −3.26 points, 80% CI [−7.3, 0.8]). When the missing data was imputed, the result did not change (t = −.71, p = .48)
Abbasi et al. 2017, Iran, RCT	Intervention (Software) N = 50 Intervention (Booklet) N = 51 Control N = 52	Both intervention groups had explanation on how to use them, the educational content was explained, and they were asked to study the booklet and software until a sufficient mastery was obtained on the educational content of software and booklet. Participants were called by the researcher every week to remind them to study the training booklet and software. Interventions contained similar content on: situation modification in pregnancy, stretching exercises and breathing, breathing pattern, relaxation, and massage.	F2F One 30-minute session 30-34 weeks gestation Midwife	Routine antenatal care and education Not described further.	**Knowledge about pain management** Questionnaire of 10 questions created by researcher. One point for every correct answer.	By adjusting the baseline score, the mean score of knowledge after the intervention in both software group (mean difference = 5.5; CI 95%: 4.6 to 6.3) and booklet group (3.4; 2.5 to 4.2) was significantly higher than the control group. Also, the increase in knowledge score in the software group (2.1; 1.2 to 9.2) was significantly higher than the booklet group
Ip et al., 2008, Hong Kong, RCT	Intervention N = 60 Control N = 73	**Self-Efficacy Enhancing Educational Programme (SEEEP) based on Bandura’s self-efficacy theory** Demonstration of coping behaviours, including breathing and relaxation, distraction, and cognitive restructuring of pain, to help participants control emotional tensions and pain during labour. Biopsychological phenomena of childbirth; strategies of coping with childbirth discomfort and its relationships with self-efficacy for childbirth. Returning demonstration of the taught coping skills by the participants. Vicarious observation of role models in a VCD depicting how two Chinese pregnant mothers modelled perseverant success in reinstating control over childbirth with the use of coping behaviours. Making a verbal contract to rehearse the taught coping skills with the use of mastery aids at home between and after sessions Given pamphlet summarising the main coping methods for guiding self-practice and a practice log for daily entries as a means of self-monitoring of their successful practice efforts in the hope that a sense of control was built through structured practice in the exercise of control over challenging conditions Verbal persuasion through positive encouragement.	F2F group sessions Two 90-minute sessions Nurse	Usual care Physical check-up and attending childbirth classes on voluntary basis.	**Retrospective perceived labour pain intensity** VAS 10 cm line “no pain at all” to “unimaginable pain”	Intervention group reported reduced perceived labour pain intensity (p < 0.01, early stage and p = 0.01, middle stage)
Firouzbakht et al., 2015, Iran, Quasi-experimental	Intervention N = 63 Control N = 132	Physical and anatomical changes during pregnancy, psychological health, warning symptoms during pregnancy, the pros and cons of vaginal and caesarean delivery, stages of delivery, breastfeeding, and family planning theoretical training Consultations of 15 min long in forms of questions and answers. Mental and muscular exercises, training proper positions during labour and delivery, proper breathing during pregnancy, labour, and delivery, and 30 min of practicing for pregnant women	F2F plus audio-visual Eight 90-minute sessions Four doulas	Routine prenatal care Not described further.	**Labour pain intensity: collected during labour** VAS 0-100	Intervention group reported reduced perceived labour pain intensity. Intervention group: 85.68 (1.85) Control group: 90.99 (14.72) (P = 0.03)
Mahnaz et al. 2019, Iran, RCT	Intervention N = 36 Control N = 34	Presentation covering all objectives and including photos and posters followed by Q&A. Awareness of physiologic changes in the body during pregnancy, such as breast enlargement, nausea, fatigue, pelvic inflammation, and abdominal and perineal changes, changes in orgasm patterns during sexual activity in this period, cultural and religious restrictions affecting women's sexual function in pregnancy, awareness of sexual function changes in the third trimester of pregnancy, using sexual positions with fewer problems, avoiding sexual activity in case of uterine contractions and vaginal bleeding (i.e., see a doctor as soon as possible, avoiding sexual activity in case of an incompetent cervix, prevention of sexually transmitted diseases In addition, an educational booklet written in simple language was provided for the pregnant women to study at home with their husbands. During the 4 weeks after the last session, the intervention group received no further contact or additional information (other than the cases provided in the educational pamphlet)	F2F group sessions Four sessions Healthcare workers	Training on benefits of breastfeeding and normal delivery. No further description.	**Sexual pain intensity** Included within questionnaire	No difference in sexual pain intensity after intervention (P = 0.78)
Ucar and Golbasi, 2019, Turkey, Quasi-experimental	Intervention N = 52 Control N = 59	Diagnosis and evaluation of the perception of childbirth, getting acquainted between educator and participants, configuration of group sessions (Aim, method, duration, frequency, etc.), sharing the beliefs and perceptions of the group members about childbirth, summary Diagnosis of fear of childbirth, feedback on the previous session, setting and sharing the agenda for the session A-B-C model of cognitive behavioural approach, A-B-C model of fear of childbirth, summary Giving “Fear diary” homework, “Severity of labour contractions diary” homework Information about childbirth and correction of misinformation; mood; feedback on previous session; factors that affect childbirth; signs of labour starting; stages of labour; delivery room and the procedures carried out in delivery room; summary Giving “Unfinished stories” homework Coping with uterine contractions; feedback about the previous session; breathing techniques used at the first stage of labour; pushing techniques used at the second stage of labour; summary Giving homework “Unfinished stories” homework; “Birth plan” homework Coping with fear of childbirth and creating a more positive perception of birth; mood; feedback on previous session; reviewing homework; creating a positive perception of birth; relaxation techniques; summary Feedback on previous session; strengthening the breathing techniques used during labour; reviewing information obtained during the group process; evaluation of the group process	F2F group classes Six 45-minute sessions over three weeks Midwives	No intervention / education No further description.	**Retrospective perceived labour pain intensity** NPRS (VAS) 0-10 “no pain” to “unbearable pain”	Intervention group reported reduced perceived labour pain intensity ((r = –0.421, p = .000)
Camlibel and Mete, 2023, Turkey Quasi-experimental	Intervention N = 30 Control N = 30	Introduction, thoughts, and expectations about the training program, emotions, and thoughts about the concept “delivery”, the underlying reasons of positive/negative emotions and thoughts about delivery, history of fear, explaining the aims of the class, “Fear-Tension-Pain” cycle, the role of the hormones in delivery, the effect of fear on the hormones needed for the delivery and on the delivery action, explaining the aims of the preparation for delivery classes, summary, muscle and relaxation exercises, assignment. Summary of previous class, sharing the assignments of the first class, the three rules that are influential in changing the viewpoint on delivery (thought, emotion and behaviour, the power of the language, motivation), the methods that may be used to ensure relaxation (breath exercises, visualisation/imaging, imagination, forming a mental area), muscle and relaxation exercises, assignment. Sharing the assignments of the second class, summary of previous class, the indications of the start of the delivery, real/fake birth pains, the stages of the delivery and its mechanism (opening, delivery, the birth of the placenta), the practices recommended to be made at home when the delivery starts, the hospital process, the delivery video, talking to the doctor and other healthcare staff about the delivery, muscle and relaxation exercises, assignment. Sharing the assignments of the third class, summary of previous class, last preparations for the delivery (delivery pack, transportation to hospital), caesarean, epidural anaesthesia, postpartum early period, delivery video or positive delivery history, relaxation exercises, ceremony for participation certificate, evaluation of the training.	F2F group sessions Four 120-minute sessions total (once weekly for four weeks) Nurses	Routine antenatal care No further description	**Labour pain intensity** Latent phase Active phase Transition phase VAS 10 cm ruler	Significant difference between the study and the control group in terms of latent phase (U = 2.260, p = 0.024), and active phase (U = 2.630, p = 0.009) pain points averages; while there was no significant difference between the transition phase (U = 1.611, p = 0.107) pain points averages.
Citak Bilgin et al., 2019, Turkey, Quasi-experimental	Intervention N = 64 Control N = 57	Introduction, the aim of the course, anatomy and physiology of reproductive organs, the role of uterus and baby during delivery, physiology of pain in birth, fear and its effects at birth, relaxation and mental imagination techniques, initial symptoms of birth Stages of birth, true and false labour pain, the effect of hormones at birth, emotional and physical support at birth, breathing techniques, communication with the baby in the womb and its importance, effects of relaxation on mother and foetus, progressive relaxation Non-pharmacological techniques used in coping with labour pain, massage techniques, positions can be used at birth, the use of a birthing ball, pushing techniques Anaesthesia at labour, caesarean section, interventions to birth, the establishment of a delivery plan Mother-baby relationship, baby’s care (bathing, dressing, etc.), breast milk and its importance, breastfeeding, breastfeeding techniques, breast problems and care, milking with hand and pump, storage of the milk, summary what has been learned in the education and handing out of certificates The participants were given music CDs and education booklets at the beginning of each session and were asked to repeat the exercises with music at home every day.	F2F Five 3-hour sessions total. (One 3-hour session weekly for five weeks) Doctor of nursing	No intervention / education	**Retrospective perceived labour pain intensity** VAS 0-10 “no pain at all” to “unimaginable pain”	Intervention group reported significantly reduced perceived labour pain intensity (p = 0.016)
Miquelutti et al., 2013, Brazil, RCT	Intervention N = 97 Control N = 100	Birth Preparation Programme (BPP): Combined physical and educational activities alongside routine antenatal care. Key components included: Pelvic floor awareness & training: Rapid (30×) and sustained (20 × , 10s) contractions, with guidance for correct sensation; included strategies to identify muscles if unsure. Education: Physiology of labour, prevention of pregnancy pain, role of pelvic floor in pregnancy, delivery, and postpartum, breathing exercises, and non-pharmacological pain management. Physical exercises: Stretching: Head/neck, trunk (anterior, posterior, lateral), lower limbs, spine/pelvis mobilisation, lumbar traction Venous return: Lower limb exercises (standing and lateral positions) Abdominal: Transverse abdominis activation Relaxation & coping techniques: Breathing exercises, progressive relaxation, massage, mentalisation Home practice and guidance: Daily exercises, encouragement for ≥30 min aerobic activity, written instructions with safety information (ACOG guidelines)	F2F 50-minutes. fortnightly between 31 and 36 weeks of pregnancy and weekly from 37 weeks of pregnancy onwards Physiotherapists, Nurses, Medical Staff	Routine antenatal care Educational activities routinely offered at the prenatal clinics: information on breastfeeding, the signs and symptoms of labour and a visit to the delivery ward. During labour, at the maternity ward, non-systematic information on the use of non-pharmacological pain relief techniques were provided by trained physiotherapy, nursing, and medical staff.	**Lumbopelvic pain** VAS 10 cm ruler	No difference in lumbopelvic found between groups
Heim and Makuch, 2024, Brazil, Quasi-experimental	Intervention N = 50 Control N = 50	All educational sessions were similar when performed in-person or virtually (by WhatsApp platform) and began with a brief conversation to encourage women to share their feelings, doubts and concerns about labour and delivery. Topics: contractions, pain during labour and delivery, and how to seek relief by adopting the upright position and using breathing techniques and relaxation. The non-pharmacological techniques were selected because they constitute a complementary approach to pain relief. Once women learn and become confident with their use, they become independent and more active in controlling their birthing process, they can be used by the woman regardless of other resources and are harmless for the baby and the mother. Breathing techniques, upright positions and relaxation techniques were performed by the pregnant women at all educational meetings. For the breathing technique, women were instructed to take a deep, nasal, slow abdominal inspiration, followed by a deep, prolonged exhalation through the mouth. The women were stimulated to maintain simultaneously global relaxation in the chosen position, mainly an upright position (‘smell the flower and blow out the candle flame at the same time relaxing your body, feel where you are tense, and relax your body’). Among the vertical and comfortable positions during labour, the following positions were trained: sitting on a birthing ball, standing, walking between contractions, squatting, and sitting, and any vertical position that women felt was comfortable and helped to control pain, to perform the breathing techniques and to relax. All the exercises were trained during a time like the duration of a contraction.	F2F + virtually (via WhatsApp) Two-five 20-minute sessions Interrupted by COVID-19 pandemic Physiotherapists, Nurses, Physicians	No intervention / education	**Retrospective perceived labour pain intensity** VAS 0-10 “no pain” to “maximum level of pain tolerated”	No difference in intensity labour pain reported between the two groups Intervention group: 8.8 (SD = 0.23) Control group: and 9.1 (SD = 0.29) (p < 0.03)
El-Kurdy et al., 2017, Egypt, RCT	Intervention N = 52 Control N = 52	Overview of Labour and Delivery: Welcome & develop rapport among group participants; explain objectives; encourage participants to express their feeling and sharing thoughts about childbirth process; introduction to pre-test questionnaires (Interview – CBSEI); anatomy and physiology of female genital organs; premonitory signs of labour; true and false labour pain; what to bring to the hospital; stages and phases of labour Coping with Labour Pain: Review content from class one; nature of labour pain; non-pharmacological coping measures with labour pain; medications used in labour Delivery and Postpartum: Brief review the content of class 1–2 and ask for the question and discuss the answer if they have; delivery variations (Episiotomy and C-section); immediate postpartum care in hospital; new-born care; post-test (CBSEI)	F2F Posters, slide presentations, animation videos, and demonstration Three 90-minute weekly sessions Midwives	Routine ANC Exposed to all conditions of intervention group except for antenatal education sessions.	**Retrospective perceived labour pain intensity** VAS 0-10 “no pain” to “most intense pain”	1st stage: antenatal education and control groups respectively (5.08 ± 0.68 & 7.40 ± 0.5). 2nd stage: antenatal education and control groups respectively (6.52 ± 0.5 & 8.56 ± 0.7). Highly significant differences between the two groups regarding the mean score of labour pain at the 1st and 2^nd^ stages of labour (p = < 0.001).
Uslu Yuvaci et al., 2021, Turkey, Pre-post-trial	Intervention Primigravid pregnant women N = 144 No control group	Antenatal education sessions consist of: physiology of labor, maternal participation in labor, coping with labor pain (breathing and relaxation exercises), cesarean section information, delivery team roles, delivery video, discussion of worries; educational materials included PowerPoint presentations, visual and written info from the Turkish Ministry of Health	F2F Three 45-min group sessions of 8–10 participants conducted by a certified midwife and nurse	No control group but pre-post comparison	Oxford Worries about Labour Scale (OWLS), including sub-dimensions: Pain and Distress, Prenatal Uncertainty, and Interventions; measured pre-education, 2 weeks post-education, and at postpartum 4–6 weeks	Post-education and postpartum OWLS scores were significantly improved compared to pre-education scores across all sub-dimensions (p < .05), indicating reduced worries about labor, pain, and interventions.
Akca et al., 2017, Turkey, Quasi-experimental	Intervention N = 77 Control N = 75	Antenatal education sessions consist of: reproductive anatomy and fetal development, antenatal care, aromatherapy, massage, prenatal exercises and yoga, stages and signs of labor, breathing and pain relief techniques, operative delivery, breastfeeding, neonatal and postpartum care	F2F group sessions (max 15 women) 4 sessions, 3 hours each, once per month Delievered by psychiatrist, dietician, obstetrician/gynecologist, sports-medicine physician, neonatologist, two nurses	No education or intervention	**Retrospective perceived labour pain intensity** VAS 0-10 “no pain” to “most intense pain”	Women who attended the program reported significantly lower pain (VAS, p = 0.01)
Karakoc et al., 2025, Turkey, Quasi-experimental	Intervention N = 64 Control N = 64	Antenatal Education Content: 3-day childbirth preparation program: Day 1: Philosophy of birth, fear of childbirth, pelvic anatomy, uterine muscles, hormones Day 2: Birth plan, normal birth, procedures during birth Day 3: Puerperium, breastfeeding, newborn care	F2F group sessions with lectures and discussions Midwifes	No education or intervention	Pain during labor was assessed retrospectively indirectly via the pain period subdimension of the Perception of Birth Scale (5 items, part of the 25-item scale). Scores reflect the mother’s perception of pain experienced during labor; higher scores indicate a more positive experience (lower perceived pain).	Women who received prenatal education had significantly more positive pain experiences (P ≤ 0.001). Logistic regression showed that lower education, negative pain experiences, and lower awareness were associated with not receiving prenatal education.

**Abbreviations:** ANC, antenatal care; F2F, face-to-face; NPRS, numerical pain rating scale; RCT, randomised controlled trial; VAS, visual analogue scale

**Table 4:** Data extraction table for qualitative studies.

Author; Year; Country; Study design	Participants	Antenatal education content, delivery method, and profession	Data collection method	Analytic approach	Key themes and pain related findings
Hassanzahed et al., 2021, Iran, Qualitative study	Participants who participated in the antenatal education sessions N = 13	Eight-session, midwife-led childbirth preparation programme based on Ministry of Health recommendations. Sessions (90 minutes each) combined theory and practical components, including stretching, relaxation, massage, and breathing techniques to support labour preparation and pain coping. Programme delivered face-to-face in group settings and provided free of charge.	In-depth semi-structured interviews lasting about 40–60 minutes with women 1 month after birth	Conventional content analysis	Six themes were identified: incentive and learning about pregnancy and childbirth, active participation in labour, sense of self control,use of non-medical pain relief methods during labour, preferring vaginal birth over caesarean section, and positive childbirth experience. Women attributed their use of non-medical pain relief during labour to participation in childbirth preparation classes. Breathing techniques, massage, and movement exercises learned during the programme were perceived as effective in reducing labour pain, promoting relaxation, and improving pain tolerance. These strategies enhanced women’s sense of control and active participation during labour.
Miquelutti et al., 2013, Brazil, Qualitative study	Women who participated in antenatal education (N = 11) Vs Women who did not participate in antenatal education (N = 10)	Birth Preparation Programme (BPP): Combined physical and educational activities alongside routine antenatal care. Led by physiotherapists, nurses, and medical staff. Key components included: Pelvic floor awareness & training: Rapid (30×) and sustained (20 × , 10s) contractions, with guidance for correct sensation; included strategies to identify muscles if unsure. Education: Physiology of labour, prevention of pregnancy pain, role of pelvic floor in pregnancy, delivery, and postpartum, breathing exercises, and non-pharmacological pain management. Physical exercises: Stretching: Head/neck, trunk (anterior, posterior, lateral), lower limbs, spine/pelvis mobilisation, lumbar traction Venous return: Lower limb exercises (standing and lateral positions) Abdominal: Transverse abdominis activation Relaxation & coping techniques: Breathing exercises, progressive relaxation, massage, mentalisation Home practice and guidance: Daily exercises, encouragement for ≥30 min aerobic activity, written instructions with safety information (ACOG guidelines)	Data was collected via semi-structured interviews lasting between 30–40 minutes. Participants were invited and data was collected between 24–48 hours after the birth and before discharge.	Thematic analysis	Three themes were identified from the interviews: control of labor, positions adopted during labor, and satisfaction with labor. Women who participated in the systematic BPP reported that the knowledge and exercises learned during the programme helped them feel more in control during labor and better able to manage pain and anxiety. Upright positions (sitting, standing, walking, using the birthing ball, shower) were perceived as more comfortable and effective in reducing pain and facilitating mobility, while horizontal positions were associated with increased discomfort. Participants used breathing, pelvic floor, and movement exercises to cope with contractions and reported that these strategies enhanced their sense of control and confidence. Women who did not participate in the BPP also found upright positions helpful but often required guidance from staff and felt less confident to move independently. Overall, satisfaction with labor was linked to perceived pain control: those who effectively used pain-coping strategies reported higher satisfaction, whereas excessive pain, prolonged labor, or instrumental interventions (e.g., forceps) were associated with lower satisfaction.

### Mode of antenatal education

Among the included studies, antenatal education was delivered in three of the following way: face to face, a hybrid of in person and online, and online only. 14 studies were solely face-to-face [23–28,31–33,35–39]. Two studies used a combination of face-to-face and virtual online materials [30,34]. One study utilised virtual online materials only which included audio-video media through a social networking application [29].

### Frequency and duration of antenatal education

The frequency and duration of antenatal education sessions varied widely across all included studies. The length of the sessions varied from 20 minutes [34] to six hours [26]. Total time spent in antenatal education sessions ranged from 30 minutes [25] to 18 hours [26]. Two studies reported the same total time spent on antenatal education, 4.5 hours [27,32] while all remaining studies differed in total time [23–26,28–30,33–39]. Three studies reported their sessions were 90 minutes in length [23,27,30]. The number of antenatal education sessions ranged from one [25], to eight sessions [29,30]. Frequency of sessions ranged from daily for three days [26] to fortnightly [24].

### Profession(s) delivering the antenatal education

In 12 studies, only one profession delivered the antenatal education [23,25–33,36,38] whereas in the other five studies, a combination of professions delivered the education [24,34,35,37,39].

Nurses delivered antenatal education in three studies [23,29,33] while midwives delivered in five studies [25,27,32,36,38]. In one study antenatal education was delivered by both nurses and midwifes [37]. Doulas [30], a Doctor of Nursing [31], ‘mindfulness instructors’ [26] and ‘healthcare workers’ [28], each delivered the intervention in one study respectively. A combination of healthcare staff including physiotherapists, nurses, physicians, midwifes, dietitians etc. delivered the antenatal education in four studies [24,34,35,39].

### Control group among included studies

15 out of 17 studies included in the review had a control group. Only one interventional study was a pre-post-trial without a control group [37] and one qualitative study did not have a control group which received a routine antenatal care [38]. The control groups in eight of the reviewed studies received routine antenatal care [23–27,29,30,33]. Six control groups received no education nor intervention [31,32,34–36,39]. Mostly, control participants attended general antenatal sessions that covered basic pregnancy care, foetal development, and signs of labour, but did not specifically focus on pain management techniques [27,29]. Participants in the control group of one study received training on the benefits of breastfeeding and normal delivery [28].

### Pain outcome measures

Nine of the 15 included interventional studies measured labour pain intensity using a visual analogue scale (VAS) [23,26,27,30–35]. Eight of these studies measured labour pain retrospectively [23,26,27,30–32,34], while one measured during labour [33]. Additional outcome measures used included: labour pain duration [30]; knowledge on pain management using a self-produced questionnaire [26]; sexual pain intensity postpartum [29]; pain catastrophising scale in labour [27]. One study measured postpartum pain, namely dyspareunia [29]. One study used Perception of Birth Scale [36] while another one used Oxford Worries about Labour Scale [37] both of which has pain as sub-domains. Two qualitative studies focussed on subjective pain experiences, coping skills, and pain related birth satisfaction [38,39].

### Content of antenatal education packages targeting pain

Of the 15 included experimental and two qualitative studies, interventions/antenatal education programs were described to varying levels of detail. Generic and varied pain-related topics were listed in twelve studies [23,24,26,27,29,31,33–35,37–39] whilst five did not mention pain [25,28,30,32,36]. Only one of seventeen studies explicitly stated a proposed mechanism by which their intervention addressed pain [29], proposing their birth preparation program “may reduce anxiety and fear of childbirth and increase self-efficacy and confidence by offering knowledge and practical coping skills. This may influence the perception of labour pain and enhance birth experience and satisfaction” [29].

Antenatal education interventions covered a range of pain-related topics aimed at preparing pregnant women for labour and postpartum pain management. Four interventions included the distinction between “true and false labour pain”, helping participants recognise the signs of active labour [27,29,31,33]. Only one study reported including education on pain physiology [31]. Cognitive restructuring and self-efficacy training were also introduced in some programmes [23,32].

Non-pharmacological pain management techniques were commonly included in conjunction with antenatal education, such as breathing techniques, relaxation techniques, pelvic floor exercises, massage, and a birthing ball. Nine studies referred to breathing exercises and relaxation techniques within their intervention to help manage labour pain [24,25,29–35,37,39]. Five studies reported utilising massage [24,25,31,35,38]. Two studies reported using a birthing ball [31,34]. One study reported teaching pelvic floor exercises to facilitate pain reduction and improve comfort during labour [24].

## Discussion

This scoping review aimed to map the contents and characteristics and gaps of current antenatal education programmes that target pain management during pregnancy, labour, and the postpartum period, including women’s lived experiences. In doing so, it aimed to highlight gaps in the literature to inform future research and enhance clinical practice, ultimately improving maternal and neonatal health outcomes while reducing strain on healthcare systems. This scoping review identified 17 studies exploring antenatal education programmes targeting labour and postpartum pain, published between 2008 and 2025 across seven countries. Distinguishing between true and false labour pain was the most frequently reported topic. Most studies employed face-to-face delivery, with a minority using virtual or hybrid formats. There was substantial variability in session frequency, duration, and total exposure time. Education was most often delivered by a single professional, though multidisciplinary approaches were also noted. Control groups primarily received routine antenatal care, with limited focus on pain-specific education. Nine studies measured labour pain intensity using a VAS, predominantly retrospectively. Only one study articulated a clear mechanism linking education to pain outcomes, and only one study incorporated elements of pain neuroscience education. Techniques such as breathing, relaxation, massage, and cognitive strategies were used inconsistently across interventions. Importantly, qualitative studies highlighted women’s perceived gains in pain coping, self-efficacy, and sense of control, offering insight into experiential mechanisms that were largely absent from quantitative trial reports.

### Themes

Despite each of the included studies measuring a pain-specific outcome, only 12 of the 17 studies mentioned pain when describing the intervention. Furthermore, none of these 12 studies provided a detailed description of the education content specific to pain-management such that the programme could be replicated. Descriptions were often limited to broad statements such as “information was provided on the prevention of pain in pregnancy” [24]; “nature of labour pain” [27]; “Fear-Tension-Pain cycle” [33]. This lack of detail suggests either a limited depth in understanding of pain-related topics, absence of consensus about which pain-related topics should be prioritised within antenatal care, or both. On the other hand, qualitative studies provided richer descriptions of how specific techniques such as breathing, massage, movement, and pelvic floor awareness were learned, practiced, and applied during labour, and how these contributed to perceived pain coping and control [38,39]. Antenatal education services remain largely unregulated, and academics and clinicians in this area appear to share the view that these services should be standardised across healthcare settings [14,40]. Findings from this review reinforce the need for consistent, clearly defined, and reproducible pain-specific content within antenatal education programmes to optimise maternal outcomes.

The topic of distinguishing between “true and false labour pain” was reported in four of the included studies [27,29,31,33]. True labour pain may be defined as regular, increasing uterine contractions that cause progressive cervical dilation and lead to childbirth, whereas false labour pain can be categorised as irregular, non-progressive contractions that do not cause cervical changes and do not result in labour. Misinterpreting false labour as true labour may increase fear and anxiety, particularly among nulliparous women, potentially contributing to unnecessary obstetric interventions [41]. Heightened fear of childbirth is identified as a common reason for caesarean delivery [42], and thus a deterrent from pre-existing plans for physiological birthing. Furthermore, unnecessary hospital visits may place additional strain on midwives and the health system, potentially detracting from their care of women in true labour [43]. These reasons may partially explain the emphasis of this topic within the included interventions. Conversely, there was limited focus of antenatal education on pain self-management techniques during labour. NICE guidelines [44] recommend the use of water, TENS, and music during intrapartum care, however none of these interventions were discussed in the included studies. However, qualitative findings suggest that when women are taught practical coping strategies alongside labour education, they feel more confident in managing contractions and remaining active during labour [38,39]. Future antenatal education interventions should therefore integrate labour recognition education with structured, conservative pain-management strategies.

This review found an absence of explicitly stated mechanisms by which pain was managed. Similarly, only one study reported including the topic of pain physiology [31]. Inferred mechanisms across the remaining studies were predominantly psychological, such as enhancing self-efficacy, reducing fear, and improving coping. Contemporary understanding of pain science has significantly developed in the last two decades. The recency of such developments, and a growing dependence on medicalised birth [45], may suggest this knowledge of pain has not yet been understood, adopted, nor incorporated by midwives and other professionals involved in labour pain-management. This gap may help explain the absence of pain physiology in the included studies, as well as the lack of proposed mechanisms of action. Qualitative studies in this review implicitly supported psychological mechanisms, with women describing increased control, confidence, and active engagement in labour as central to pain coping [38,39]. Understanding the neurophysiological mechanisms of pain modulation has proven effective in reducing rates of medicalised birth: in their meta-analysis of nonpharmacologic approaches for labour pain management, Chaillet et al. [11] highlighted an association between interventions based on central nervous system control, including pain education, significantly reduced rates of caesarean delivery and pain medication usage. This prompted the 2018 Canadian Obstetric Guideline No. 355 that recommended educating staff on neurophysiology of pain perception to increase physiological birth rates [46]. These findings underscore the need for greater integration of pain science and explicit mechanistic frameworks within antenatal education.

Of the included studies, seven explored labour pain intensity with a version of the VAS, while only one included a postpartum pain-outcome, dyspareunia. The VAS is a psychometric measure of subjective pain experience used widely within research and clinical practice [47]. Among other benefits, the VAS is supported for its simplicity and versatility. However, the VAS reduces pain to a unidimensional experience, which conflicts with modern understanding of pain as a complex and multidimensional phenomenon [48]. Further, most studies captured pain retrospectively potentially introducing recall bias. In contrast, qualitative studies captured experiential dimensions of pain, including perceived control, coping strategies, and satisfaction with labour, highlighting aspects of pain not adequately reflected in intensity scores alone [38,39]. Therefore, future research would benefit from incorporating outcome measures that better capture both the quantitative and qualitative dimensions of labour pain [49].

Duncan et al. [26] were the only researchers to use the pain catastrophising scale as an outcome measure. Pain catastrophising behaviour throughout pregnancy has consistently been associated with poor labour and postpartum outcomes. These include perineal trauma and persistent perineal pain following vaginal delivery [50,51]; postpartum depression; poor social adjustment after delivery [52]. These emotional and social aspects of postpartum health are important as they have been shown to contribute significantly to a woman’s experience of pain [53] alongside socioeconomic status and culture backgrounds and beliefs [44]. Qualitative findings from this review align with this literature, as women described how antenatal education enhanced confidence, reduced fear, and supported active coping during labour-factors known to mitigate catastrophising tendencies [38,39]. Increasing women’s knowledge of pain has been shown to reduce pain catastrophising scores [54]. Interventions utilising pain neuroscience education during pregnancy have shown improvements in pain knowledge, birth self-efficacy, labour coping and lower rates of caesarean delivery and epidural use [55,56]. Therefore, to target pain catastrophising and other positive birth outcomes, it is recommended that future experimental studies develop and refine pain neuroscience education for labour and postpartum pain management.

Face-to-face delivery was the predominant mode of antenatal education across included studies. In-person clinics often include physical examinations alongside education, which cannot be completed virtually. Primiparous women across cultural and socio-economic backgrounds report a preference for in-person sessions to facilitate asking questions and connecting with other expectant parents [57]. However, the COVID-19 pandemic initiated a cultural shift towards telehealth, with its use increasingly prevalent [58]. An integrative review of women’s experiences of online antenatal education identified flexibility as a key benefit, accommodating geographical barriers, illness, and complex social situations [59]. Additional advantages included enhanced comprehensibility, the ability to revisit content, timely information delivery, and broader accessibility. Despite this, those with lower e-health literacy may face difficulties accessing digital information, contributing to a ‘digital divide’ [59]. Pregnant women are known information seekers, yet challenges exist regarding the trustworthiness of online sources [60,61], with many women citing difficulty distinguishing reliable from false pregnancy and labour information [62]. When reliable, evidence-based information is used and delivered effectively, digital education has been shown to play an important role in antenatal care. A recent study evaluated a mobile application that included pain neuroscience education combined with mindfulness training to prepare for birth [56]. A large sample size was studied and improved pain knowledge, improved self-efficacy in birth as well as lower rates of caesarean birth and epidural use were demonstrated as a result of the intervention. Such findings considered alongside two studies included in this review which used a hybrid approach [30,34], that included online resources, point to the potential innovative digital modes of care provision provided quality control measures to ensure rigor are fulfilled.

There was wide disparity in the frequency and duration of antenatal education interventions, ranging from one 30-minute session to five three-hour sessions. Pain-specific content formed only a portion of each session, meaning actual time spent on pain education was less than stated. No included country currently has mandated recommendations on the number, frequency, duration, or timing of antenatal sessions. This heterogeneity may reflect that clinical delivery is not meeting current guidelines based on service pressures [63]. While any antenatal education was linked to reduced pain medication use and increased physiological birth, these outcomes improve with more sessions, although it remains unclear if this trend eventually plateaus [64]. Delivery varied across studies: midwives and nurses led the majority, despite pain education representing less than 1% of programme hours in their training [65]. Conversely, physiotherapy courses included the most pain-related content, suggesting pelvic health physiotherapists may be well-placed to deliver pain-specific education. One study used doulas, non-medical professionals who have demonstrated effectiveness in reducing labour pain, anxiety, and caesarean rates [66]. Despite lacking medical training, doulas may offer a more cost-effective solution and help reduce health disparities [67]. Which profession is best placed to deliver pain-related antenatal education remains unclear and warrants expert consensus.

Analysing pain outcomes highlighted a stark lack of focus on the postpartum period. This may be reflective of the lack of support for women beyond childbirth [6,67,68]. In many countries, six weeks after delivery, responsibility for maternal care shifts from the midwife to the woman’s general practitioner or other services. Unfortunately, poor maternal health after this period is either being missed or is costing healthcare systems more because of higher healthcare utilisation for conditions such as persistent postpartum pain and depression, despite many cost-analyses proving the financial worth of screening for such conditions [69,70]. Although qualitative studies in this review primarily focused on labour experiences, women’s emphasis on confidence, coping, and satisfaction suggests that antenatal education may have longer-term implications for postpartum pain adjustment [38,39]. This represents a clear opportunity for future research to examine the role of antenatal education in preventing persistent postpartum pain.

### Strengths and limitations

Firstly, this scoping review involved a comprehensive and systematic search across twelve major electronic databases, ensuring a broad capture of relevant literature. To our knowledge, this is the first scoping review to specifically map the content and characteristics of antenatal education programmes focused on pain management through pregnancy, labour, and the postpartum period. The use of rigorous methodology, including adherence to PRISMA-ScR and JBI guidelines, independent and blinded screening by multiple reviewers, and efforts to contact authors for missing data, further enhances the robustness and credibility of the findings. Additionally, the focus on programme content, delivery, dosage, and pain outcomes addresses a critical gap in maternal health education and may help inform future practice and research

One limitation of this review is the reliance on authors’ descriptions of antenatal education interventions, which may have been incomplete or imprecise. Additionally, heterogeneity in study design, populations, and outcome measures limits direct comparison across studies. Although qualitative studies were included, they were few in number and varied in timing and focus, which may restrict the generalisability of experiential findings.

### Implications for future research

This review highlights a need for high-quality, theory-informed studies that evaluate antenatal education interventions specifically targeting pain management during labour and the postpartum period. Future research should integrate pain neuroscience education tailored to pregnant populations, clearly articulate mechanisms of action, and employ multidimensional outcome measures that capture both pain intensity and lived experience. Greater attention to postpartum pain outcomes is urgently required. Research should also explore optimal dosage, delivery modes, and professional roles, informed by qualitative insights into confidence, control, and coping. There is a clear need for agreement on core pain-related educational domains and delivery principles to support the development of consistent, standardised, and evidence-based antenatal education programmes.

## Conclusion

This scoping review mapped the content and structure of antenatal education interventions that target labour and postpartum pain, including both outcome-focused and experiential evidence. Overall, antenatal education programmes contained limited and inconsistently reported pain-related content. The most addressed topic was the distinction between “true and false labour pain”, while education on pain mechanisms and structured pain self-management strategies was largely absent. Only one study explicitly included pain physiology, and postpartum pain outcomes were underrepresented across the literature.

Findings from both interventional and qualitative studies highlight the potential value of antenatal education in shaping women’s confidence, sense of control, and use of non-pharmacological pain coping strategies during labour. However, the lack of clearly defined educational content, articulated mechanisms of action, and multidimensional pain outcomes limits interpretation and reproducibility. Collectively, these findings reinforce the need for targeted, theory-informed antenatal education that addresses labour and postpartum pain through conservative, non-pharmacological approaches.

An improved understanding of pain mechanisms, encompassing neurobiological and biopsychosocial processes, has been shown in other populations to mitigate pain catastrophising, reduce fear-related responses, and enhance self-efficacy. Incorporating pain neuroscience education principles into antenatal education may therefore offer a promising avenue to support labour and postpartum pain management. By providing pregnant individuals with evidence-based understanding of pain, such approaches may reduce pain-related distress and promote active coping. Further research is needed to develop, implement, and rigorously evaluate pain-focused antenatal education interventions tailored to antenatal care settings.

## Supporting information

S1 Checklist Preferred reporting items for systematic reviews and meta-analyses extension for scoping reviews (PRISMA-ScR) checklist. (DOCX)
